# A centriolar FGR1 oncogene partner-like protein required for paraflagellar rod assembly, but not axoneme assembly in African trypanosomes

**DOI:** 10.1098/rsob.170218

**Published:** 2018-07-25

**Authors:** Jane Harmer, Katie Towers, Max Addison, Sue Vaughan, Michael L. Ginger, Paul G. McKean

**Affiliations:** 1 Faculty of Health and Medicine, Division of Biomedical and Life Sciences, Lancaster University, Lancaster LA1 4YQ, UK; 2 Department of Biological and Medical Sciences, Faculty of Health and Life Science, Oxford Brookes University, Gipsy Lane, Oxford OX3 0BP, UK; 3 Department of Biological and Geographical Sciences, School of Applied Sciences, University of Huddersfield, Queensgate, Huddersfield HD1 3DH, UK

**Keywords:** basal body, ciliogenesis, FGR1 oncogene partner, *Trypanosoma brucei*, cell morphogenesis, paraflagellar rod

## Abstract

Proteins of the FGR1 oncogene partner (or FOP) family are found at microtubule organizing centres (MTOCs) including, in flagellate eukaryotes, the centriole or flagellar basal body from which the axoneme extends. We report conservation of FOP family proteins, *Tb*FOPL and *Tb*OFD1, in the evolutionarily divergent sleeping sickness parasite *Trypanosoma brucei*, showing (in contrast with mammalian cells, where FOP is essential for flagellum assembly) depletion of a trypanosome FOP homologue, *Tb*FOPL, affects neither axoneme nor flagellum elongation. Instead, *Tb*FOPL depletion causes catastrophic failure in assembly of a lineage-specific, extra-axonemal structure, the paraflagellar rod (PFR). That depletion of centriolar *Tb*FOPL causes failure in PFR assembly is surprising because PFR nucleation commences approximately 2 µm distal from the basal body. When over-expressed with a C-terminal myc-epitope, *Tb*FOPL was also observed at mitotic spindle poles. Little is known about bi-polar spindle assembly during closed trypanosome mitosis, but indication of a possible additional MTOC function for *Tb*FOPL parallels MTOC localization of FOP-like protein TONNEAU1 in acentriolar plants. More generally, our functional analysis of *Tb*FOPL emphasizes significant differences in evolutionary cell biology trajectories of FOP-family proteins. We discuss how at the molecular level FOP homologues may contribute to flagellum assembly and function in diverse flagellates.

## 1. Introduction

Coupled, N-terminally located TOF-LisH motifs define a small family of eukaryotic proteins—the FOP family—members of which are required for ciliogenesis in flagellate eukaryotes, and cortical cytoskeleton organization in plant cells. Family members conserved among flagellate eukaryotes are FOP (*standing for* FGFR1 oncogene partner), OFD1 (*mutated in* orofaciodigital syndrome 1) and FOR20 (*or* FOP-related protein of 20 kDa) [1–8]. With regard to localization, in animal cells, OFD1, FOR20 and FOP are all found at the base of cilia associated with basal bodies (or centrioles), either at the level of the triplet microtubule barrel or the transition zone. The microtubule axoneme (the defining structure of all eukaryotic flagella or cilia) extends from the basal body; the transition zone defines the most proximal region of the flagellum and exhibits its own particular architecture, where Y-shaped projections link axoneme outer-doublet microtubules to the flagellar membrane [9]. FOP, OFD1 and FOR20 are all required for the formation of a primary cilium, assembled by many types of animal cell in response to appropriate environmental cues [10]. Roles for FOR20 and OFD1 in cilia assembly have been described in the ciliates *Paramecium tetraurelia* and *Tetrahymena thermophila* [5,11,12], but we are not aware of any reports regarding functional studies of candidate OFD1 and FOP orthologues in other flagellate protists or fungi.

One member of the FOP protein family is also conserved in at least one group of aflagellate eukaryotes, land-plants. The protein TONNEAU1 (or TON1), which is most similar to FOP, interacts with at least one classic protein found at microtubule organizing centres (MTOCs), centrin, and is required for organization of cortical microtubules during cell elongation and division [13]. The absence of TONNEAU1 from *Arabidopsis*, an aflagellate angiosperm, or the evolutionarily more basal bryophyte moss *Physcomitrella patens*, which deploys flagellate motile sperm for reproduction, results in organelle mis-positioning and defective development [13,14]. Another group of organisms in which a cortical-based microtubule cytoskeleton exerts an overarching and relatively well understood effect on cell morphogenesis and division are the flagellate trypanosomatids [15–17]. Long known as the aetiological agents of a variety of serious tropical diseases (e.g. African sleeping sickness, Chagas disease, leishmaniasis), the parasitic trypanosomatid family belong to the excavate group of protists, which is widely recognized as evolutionarily divergent in comparison with other eukaryotic groups [18,19].

Flagellum assembly and function has been widely studied in trypanosomatids; many facets of this biology are conserved with other flagellate eukaryotes, but there are also notable differences. Visually, the most notable difference is the presence of a complex paraflagellar rod (PFR). Within the flagellar compartment, trypanosomatids and their nearest relatives build an elaborate extra-axonemal PFR structure, consisting of a trilaminar lattice composed of proximal, intermediate and distal domains [20]. The PFR is attached to outer-doublet microtubules 4–7 of a canonical ‘9 + 2’ microtubule axoneme and runs alongside the axoneme for much of the length of the flagellum. The PFR is essential for flagellar beating and thus cell motility [21], and in *Trypanosoma* species, PFR assembly is required for attachment of the flagellum to the cell body, which in turn is required for normal cell morphogenesis [22,23]. In *T. brucei*, the PFR is present and attached to the axoneme from the point where the flagellum exits the cell body (approx. 2 µm distant from the basal body); however, structural and/or molecular cues that define assembly and asymmetric PFR attachment are not understood.

At a molecular level, in addition to obvious proteomic differences relating to PFR assembly, there are examples of conserved proteins which have an essential flagellum assembly role that are somewhat different in trypanosomatids; for instance, the tubulin cofactor C (TBCC) domain-containing protein RP2 [24,25]. Here, the catalytic TBCC domain of the trypanosome RP2 protein uniquely lies downstream of twinned TOF-LisH motifs essential for centriolar targeting of FOP family proteins [24], whereas human RP2 is targeted to the basal body by a post-translation *N*-acyl modification [26]. As part of our ongoing studies looking at the role of *Tb*RP2 in *T. brucei* flagellum assembly, we turned our attention to the function of other trypanosomatid proteins that encode N-terminal TOF-LisH motifs. We report here the conservation of OFD1 and FOP-like proteins in trypanosomatid protists, and our unexpected observation that a *T. brucei* FOP-like protein is essential for assembly of the extra-axonemal PFR, but not the axoneme itself. Our data illustrate unexpected functional and evolutionary diversity in the role of conserved centriole-targeted proteins in eukaryotic flagellum assembly and function.

## 2. Material and methods

### 2.1. Cell culture and transfection

Procyclic *T. brucei* (927Smox [27] and S427) were cultured in SDM-79 medium supplemented with 10% v/v fetal bovine serum and haemin [28]. Constitutive expression of YFP- or GFP-tagged proteins and RNAi experiments were performed in 927Smox cells, whereas myc epitope-tagged protein was constitutively expressed in a 427 genetic background. Logarithmic phase cells were transfected and stable transformants selected using 10 µg ml^−1^ blasticidin (following transfection with pENT6B-derived endogenous tagging plasmids), 50 µg ml^−1^ hygromycin (following transfection with pPOT endogenous tagging DNA or pDEX377-derived expression plasmids) or 3 µg ml^−1^ phleomycin (following transfection with p2T7_177_-derived RNAi plasmids) [29,30]. For the routine culture of 927Smox, 2 µg ml^−1^ of puromycin was used. Transgenic cultures were kept free of selectable markers for at least 48 h prior to the start of experiments. RNAi was induced by the addition of doxycycline to a final concentration of 1 µg ml^−1^.

### 2.2. Plasmid constructs

Fusion proteins were expressed using pEnT or pDEX-based vector systems [30] or the PCR only tagging approach (pPOT [29]). For constitutive expression of N-terminal YFP tagged *Tb*FOPL, *Tb*OFD1 and *Tb*FLAM3 [31] from endogenous chromosomal loci, DNA sequences corresponding to open reading frames (orf) and 3′ intergentic regions (igr) were amplified by PCR. Resultant amplicons were digested by XbaI/XhoI (orf) and XhoI/BamHI (igr) prior to three-way ligation into Xba1/BamHI digested pEnT6B-Y. Plasmids were linearized with XhoI prior to transfection. For constitutive expression of C-terminal GFP-tagged *Tb*FOPL from endogenous chromosomal loci, pPOTv2 plasmid DNA was PCR amplified [29] and the resultant amplicon used directly for transfection.

For expression of a myc epitope-tagged *Tb*FOPL, the coding sequence minus stop codon was amplified using a two-step PCR reaction (in order to remove an internal XhoI site in the *Tb*FOPL coding sequence), where the product of the first reaction was used as template for the second. The resultant PCR amplicon was digested with HindIII and XhoI prior to being cloned into a HindIII/XhoI-digested pDEX377-myc vector [24]. For *Tb*FOP and *Tb*OFD1, RNAi orf sequences were amplified and the resultant amplicon cloned between opposing head-to-head T7 RNA polymerase promoters in p2T7-177 vector, pre-digested with BamHI and XhoI. Both pDEX- and p2T7-derived plasmids were linearized with NotI prior to transfection. Molecular masses for YFP- and myc-tagged proteins were confirmed by immunoblotting (see Results); correct genomic integration of DNA conferring expression of *Tb*FOPL::GFP was confirmed by southern blotting (not shown).

### 2.3. Microscopy and immunoblotting

Cells were settled onto coverslips and either fixed directly with 3.7% paraformaldehyde or detergent extracted for 30 s with 1% Nonidet P40 in 0.1 M PIPES, 2 mM EGTA, 1 mM MgSO_4_, 0.1 mM EDTA, pH 6.9 prior to fixation. Fixed cells were placed in methanol for 10 min prior to rehydration in PBS. Indirect immunofluorescence using polyclonal antiserum raised against recombinant *Tb*RP2 [24] and monoclonal antibodies L8C4 and L3B2 (recognizing PFR and FAZ, respectively [32]), YL1/2 [33] and anti-myc was performed as described previously or as stated in the manufacturer's instructions (myc; AbCam). Images were captured using an Applied Precision DeltaVision microscope with a Roper Scientific Photometrics Cool SNAP HQ camera at 60× magnification and processed using associated SoftWorx software and Adobe Photoshop. Nuclei and kinetoplast counts of L8C4 and DAPI labelled cells were determined using a Leica DM RXA2 microscope and associated FW4000 software.

Protein samples were separated by SDS-PAGE and immunoblotted onto Hybond P membrane (Amersham Biosciences) using standard protocols. Membranes were probed with monoclonal antibodies BB2 [34] to detect YFP::*Tb*FOPL or KMX1 [35] for the detection of β-tubulin as previously described. HRP-conjugated secondary antibodies were detected using Immobilon Western Chemiluminescent HRP substrate (Millipore) and BioRad XRS Imaging System.

### 2.4. Electron microscopy

Fixation was by addition of glutaraldehyde (2.5% final concentration, 5 min) to cultures. Cell pellets were re-suspended in 0.1 M PBS (pH 7.4) for 10 min, followed by 2.5% glutaraldehyde, 2% paraformaldehyde and 0.1% tannic acid in 0.1 M phosphate buffer (pH 7.0) for 2 h at room temperature. Pellets were washed with 0.1 M phosphate buffer (pH 7.0) and post-fixed in 1% osmium tetroxide in 0.1 M phosphate buffer (pH 7.0) for 1 h at room temperature. Samples were rinsed and stained *en bloc* for 40 min in 2% uranyl acetate, dehydrated in an ascending acetone series and embedded in Agar 100 resin (Agar Scientific). Thin sections were examined by electron microscopy using a Hitachi H-7650, operated at 120 kV.

### 2.5. Bioinformatics

Protein sequences were aligned by Clustal Omega [36], and the STRING database [37] was used to identify known and predicted interactions between human FOP and other proteins.

## 3. Results

### 3.1. Divergent FGR1 oncogene partner family proteins in trypanosomatids

**Figure 1.**
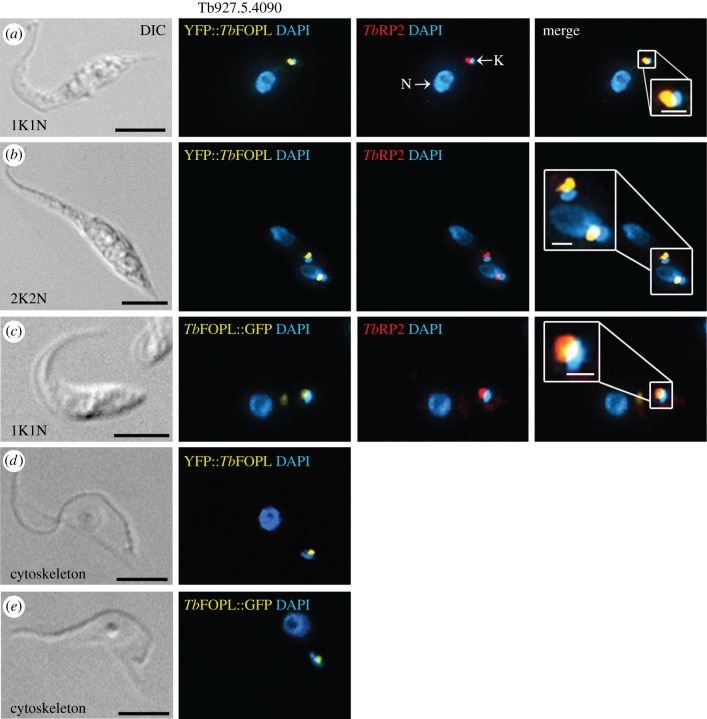
Localization of Tb927.5.4090 gene product (*Tb*FOPL) in procyclic *T. brucei*. (*a,b*) Localization of YFP::*Tb*FOPL at the mature basal body in 1K1N (*a*) and 2 K mitotic (*b*) cells. Images show detection of YFP::*Tb*FOPL relative to *Tb*RP2 in whole cells (*a–c*); insets show the same localizations at higher magnification. 6-Diamidino-2-phenylindole (DAPI) was used to detect nuclear DNA (N) and the mitochondrial genome (or kinetoplast, K). (*c*) *Tb*FOPL::GFP localization at the mature basal body in a 1K1N procyclic *T. brucei* cell; inset shows *Tb*FOPL::GFP and *Tb*RP2 localization at higher magnification. (*d,e*) Retention of YFP::*Tb*FOPL (*d*) and *Tb*FOPL::GFP (*e*) in detergent extracted cytoskeletons. Scale bars in all main panels indicate 5 µm and in the inset panels 1 µm.

Additional to *Tb*RP2, three further genes in *T. brucei* encode proteins with coupled N-terminal TOF-LisH motifs: Tb927.11.3090, Tb927.5.4090 and Tb927.10.3000. Syntenic orthologues of all three genes are present in all trypanosomatid species for which genome sequences are available at EuPathDB [38]. Tb927.11.3090 encodes an FOR20 orthologue and localizes to both pro- and basal bodies [4]. By contrast, the predicted proteins encoded by Tb927.5.4090 and Tb927.10.3000 are not immediately recognizable as orthologous to any particular FOP family protein. In that context, we also note failure to correctly predict a trypanosome FOP orthologue in both a published bioinformatics survey of centriole/basal body evolution, and within the phylogenomic co-occurrence survey that is a part of the ‘STRING’ programme [37,39]. Given the importance of a microtubule corset in defining trypanosome cell morphology, and involvement of an FOP-related protein to organizing the cortical cytoskeleton in acentriolar plant cells, we made no assumption regarding the localization of proteins encoded by Tb927.5.4090 and Tb927.10.3000. Thus, we expressed both as N-terminal fusions with YFP from their endogenous chromosomal loci (and thus under the regulatory control of the endogenous 3′ intergenic sequence; in trypanosomatids, 3′ intergenic sequences are accepted as exerting the dominant influence on gene expression). In these experiments, YFP fluorescence was compared to the indirect immunofluorescence signal obtained using polyclonal affinity-purified anti-*Tb*RP2 antibody [24]. As shown in figure 1 and electronic supplementary material, figure S1, proteins encoded by Tb927.5.4090 (figure 1) and Tb927.10.3000 (electronic supplementary material, figure S1), and tagged at the N-terminus with YFP, each co-localized with the mature basal body marker *Tb*RP2 but were not detectable at other MTOCs or other cellular locales at any point during the cell cycle.

**Figure 2.**
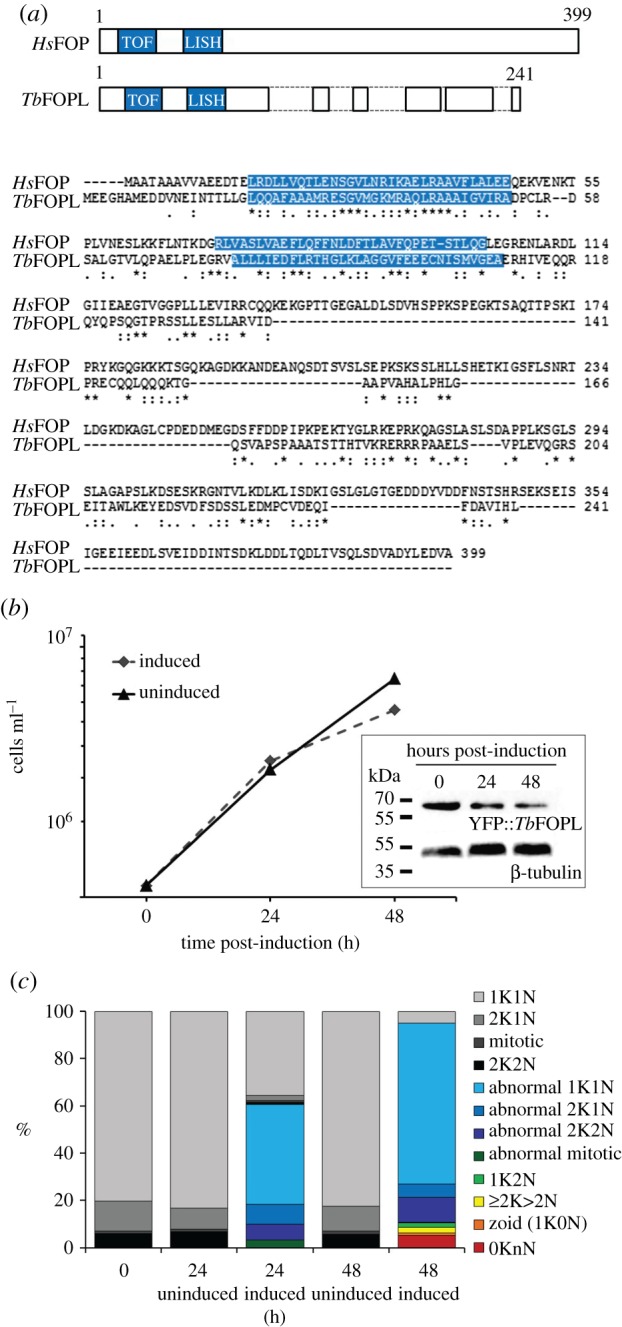
RNAi knockdown of *Tb*FOPL results in severe morphological defects. (*a*) Cartoon representation of human FOP and *T. brucei* FOPL, showing insertions necessary to achieve maximal alignment of amino acid sequences, and amino acid alignment of *Homo sapiens* FOP (accession number CAA77020.1) and *T. brucei* FOPL. (*b*) Effect of Tb*FOPL* RNAi induction on trypanosome growth (diamonds) compared to RNAi non-induced controls (triangles); immunoblotting with monoclonal antibody BB2 (detecting an N-terminal Ty-epitope) confirmed depletion of YFP::*Tb*FOPL post-RNAi induction. Anti-β-tubulin antibody KMX1 was used as a loading control. (*c*) Effect of Tb*FOPL* RNAi induction on cell morphology with regard to normal kinetoplast-nuclei number and positioning and/or normal PFR assembly.

Returning to the interrogation of *T. brucei* FOP family candidature, BLAST analyses revealed human OFD1 identified Tb927.10.3000 as the top hit, albeit with an e-value below an e^−10^ threshold and requiring the insertion of numerous gaps to produce an alignment with moderate identity and similarity. These trypanosome and human proteins also differ in length by over 200 amino acids (electronic supplementary material, figure S2*a*,*b*). Nevertheless, gene-specific RNAi provided further evidence for the *Tb*OFD1 candidature of Tb927.10.3000 (electronic supplementary material, figure S2*c*–*m*). By contrast, *Hs*FOP and divergent FOP-like proteins from *Tetrahymena* (TTHERM_00537420, or *Tt*Fop1 [40]; TTHERM_00305510; TTHERM_00689980) failed to identify candidate orthologues from trypanosomatids with expectancy values above even an e^−05^ threshold; here, both differences in size and an overall shorter protein length influence analysis outcomes. However, with the acceptance of three insertions, *Hs*FOP and the Tb927.5.4090 gene product align with reasonable identity and similarity along their length (figure 2*a*). Based on our analyses, we believe Tb927.5.4090 encodes an FOP-related protein, but following published reports of *Hs*FOP and *Tt*FOP1 [1,40], we conservatively refer to the trypanosome gene as FOP-like (or Tb*FOPL*). We also prefer the designation FOP-like because of the surprising RNAi phenotype described below.

### 3.2. *Tb*FOPL protein is required for paraflagellar rod assembly but not axoneme formation

**Figure 3.**
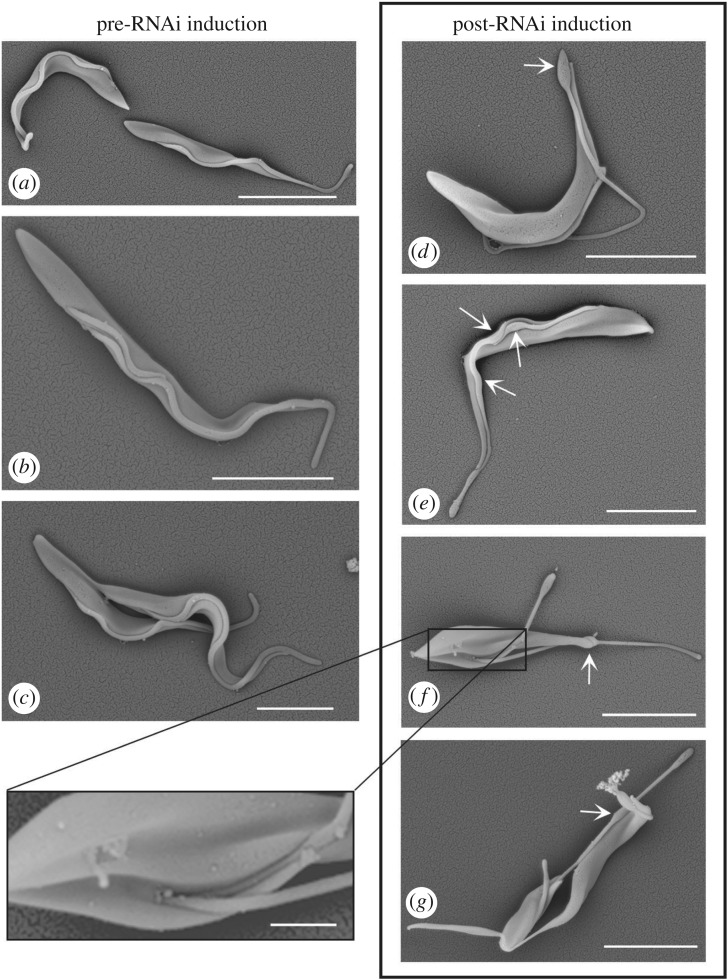
Induction of Tb*FOPL* RNAi results in flagellum assembly and cell morphology defects. (*a–c*) Scanning electron micrographs of Tb*FOPL* RNAi non-induced cells showing procyclic cells at different stages of the cell division cycle. (*d–g*) Scanning electron micrographs of Tb*FOPL* RNAi-induced cells showing distended regions within flagella (arrows), flagellar detachment and (*f*) two flagella aberrantly emerging from the same flagellar pocket, thereby illustrating one extreme of morphogenetic abnormality present. Scale bars in all main panels indicate 10 µm and in the inset panel 1 µm.

**Figure 4.**
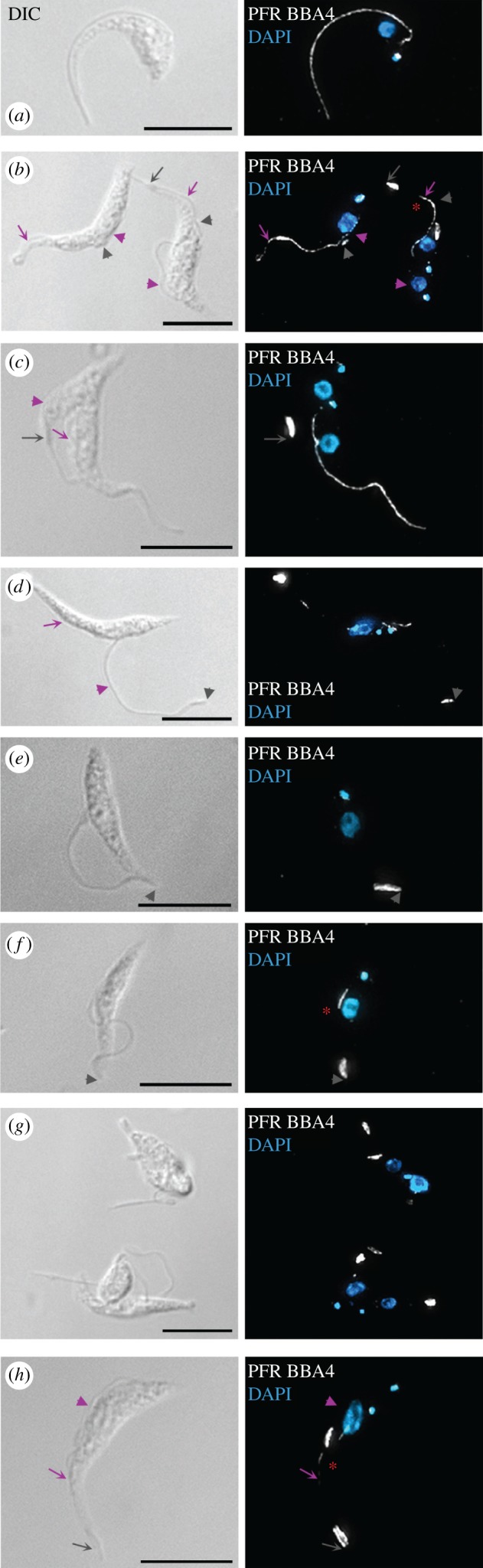
Effect of Tb*FOPL* RNAi on PFR assembly. (*a*) Immunolabelling of PFR in a Tb*FOPL* RNAi non-induced cell. (*b–h*) Immunolabelling of PFR in Tb*FOPL* RNAi-induced cells highlighting different failures in PFR assembly. The PFR-specific antibody L8C4 was used in all images to detect PFR2 protein. DAPI was used to detect nuclear DNA (N) and the mitochondrial genome (or kinetoplast, K). Grey arrows in (*b*,*c*,*h*) indicate the position of the anterior cell end; grey arrowheads indicate the distal tip of flagella, including ‘new’ elongating flagella of cells in (*b*,*d*). Purple arrows and arrowheads also denote positions of old and new flagella, respectively. Red asterisks indicate flagella where PFR assembly has apparently commenced prior to a subsequent failure. Cells in (*b*–*f*,*h*) are from 24 h post-RNAi induction; cells in (*g*) are from 48 h post-RNAi induction. Scale bars indicate 10 µm.

Following Tb*FOPL* RNAi induction, levels of YFP::*Tb*FOPL declined (figure 2*b*) and abnormal cells appeared within 24 h. By 48 h, cell growth had slowed and very few cells presented with a normal morphology (figures 2*b,c*, 3 and 4; electronic supplementary material, figure S3); cells at this stage varied in size and distinct intra-flagellar swelling was frequently observed (figure 3). Swelling was typically observed either at the very distal end of the flagellum (figure 3*d*; arrow) or within the flagellum (figure 3*e–g*; arrows). Cells decorated for immunofluorescence microscopy with the monoclonal antibody L8C4 (which detects PFR2; one of the two major proteins that form the PFR) indicated the variable width of the flagellum noted in the SEM images was the result of defective PFR assembly (figure 4). Instead of the uniform PFR2 signal seen along the length of the flagellum, from the point of cell body exit in normal cells (figure 4*a*), we typically observed cells where PFR2 signal was absent, except for the accumulation of PFR2 protein at a point coincident with the end of the cell body and/or the distal tip of the flagellum (figure 4*b–h*). We also observed cells where sometimes a faint PFR2 signal was present in the proximal region of the flagellum but the signal was then lost, indicating an initiation of PFR assembly but subsequent failure of PFR assembly within the same flagellum (figure 4*b,f,h*). In cells where the PFR of a pre-existing flagellum was fully formed, we saw no evidence for subsequent PFR loss, although assembly of PFR in new elongating flagella was perturbed (figure 4*c*). In such cells, an accumulation of PFR2 at the end of the cell body and/or flagellar tip was often evident; in some of these cells, detachment of the flagellum from the cell body was also evident in the regions lacking PFR2 (figure 4*d–g*). The representative images shown in electronic supplementary material, figure S3 illustrate how PFR formation was compromised in virtually all (more than 95%) cells 48 h post-RNAi induction. We reported previously that for calmodulin (CaM) RNAi mutants, where PFR formation also fails completely, the default status is for flagellum–cell body attachment and that flagellum detachment occurs some time later [22]. Examples of cells with attached flagella that lacked PFR were observed in RNAi-induced Tb*FOPL* mutants (figure 4*c,h*). However, by 48 h post-induction of Tb*FOPL* RNAi, considerable heterogeneity in cell morphology was evident (figure 4; electronic supplementary material, figure S3). Included in this heterogeneity were ‘cells’ or, perhaps more accurately, cell ‘slivers’ apparently lacking flagella and of varying size. These were the likely consequence of asymmetric cell division; the higher resolution afforded by SEM emphasized the irregularities in cell morphogenesis that could occur following *Tb*FOPL depletion (figure 3*d–g*).

**Figure 5.**
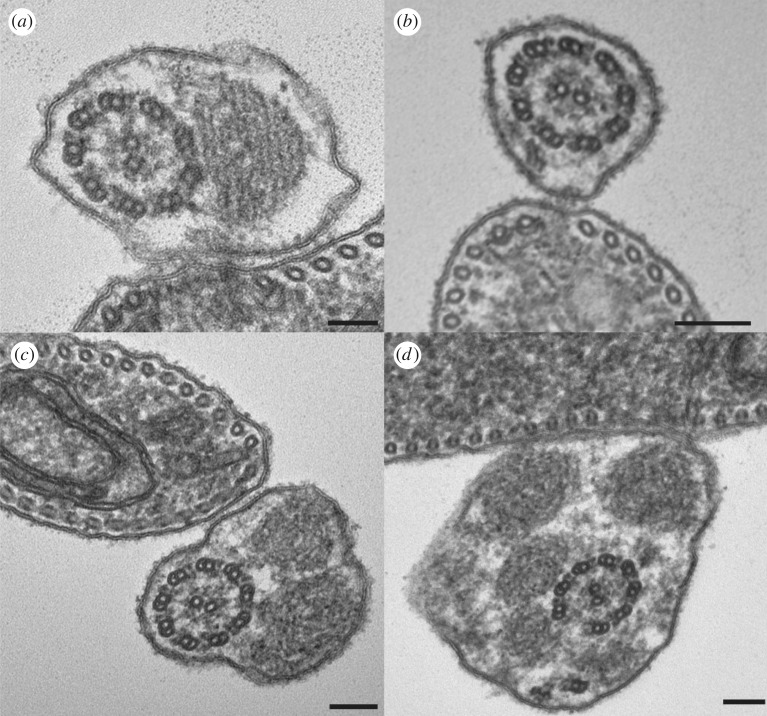
TEM of flagella following induction of Tb*FOPL* RNAi. (*a*) Transverse thin section through the flagellum of a Tb*FOPL* RNAi non-induced cell shows normal arrangement of axoneme and PFR. (*b*) The absence of PFR assembly following Tb*FOPL* RNAi; dynein ATPases, radial spokes and central pair (CP) projections are all present. (*c*) Massive accumulation of unstructured PFR material in association with an axoneme where dynein ATPases, radial spokes and CP projections are present. (*d*) A rare example of loss of axoneme integrity following Tb*FOPL* RNAi. Scale bars indicate 100 nm.

Depletion of centriolar FOP in cultured mammalian cells results in loss of ciliogenesis [6,7]. By contrast, flagellum assembly was maintained post-*Tb*FOPL RNAi induction, albeit with flagella that were often detached from the cell body. Indication that an intact axoneme was assembled within these flagella came first from comparison with *T. brucei* intraflagellar transport (IFT) RNAi mutants [41,42], and *Tb*OFD1 RNAi mutants (electronic supplementary material, figure S2). In these mutants, elongation of a ‘flagellar sleeve’ of particularly narrow diameter (approx. 70 nm, cf. axoneme diameter approx. 180–200 nm) is seen. It reflects flagellar membrane elongation in the absence of axonemal microtubule extension, and it is conceivably a consequence of the IFT-independent movement of the trypanosome ‘flagellar connector’ [41], which guides flagellum elongation, cytotactic inheritance of organelles and overall cell morphogenesis in *T. brucei* [16]. The diameter of flagella seen from SEM analysis of *Tb*FOPL RNAi mutants was indicative of IFT-dependent axoneme elongation, rather than sleeve formation. Nonetheless, mindful of the importance of FOP for axoneme formation in ciliated mammalian cells [6,7], we looked at axoneme ultrastructure in our *Tb*FOPL RNAi mutants. In 50 transverse sections (out of a total of 51 analysed), axoneme structure looked normal, irrespective of whether PFR was absent, or the axoneme partially surrounded by an accumulation of PFR protein(s) (figure 5*b,c*). This contrasts with the loss of outer-doublet and/or central pair microtubule integrity associated with *T. brucei* flagellum RNAi mutants depleted for radial spoke, central pair or nexin-dynein regulatory complex components, and selected, cultured, fixed and prepared for electron microscopy using the same protocols as the current study [43–45]. This further emphasizes *Tb*FOPL is not required for axoneme formation *per se*. Transmission electron microscopy (TEM) analysis revealed that PFR protein accumulated and assembled as an amorphous structure rather than the elaborate ordered lattice observed in normal cells (compare figure 5*a* with *b*–*d*). In the one section where a defect in axoneme structure was evident, displacement of an outer doublet microtubule (figure 5*d*) was conceivably the consequence of excessive accumulation of PFR proteins.

**Figure 6.**
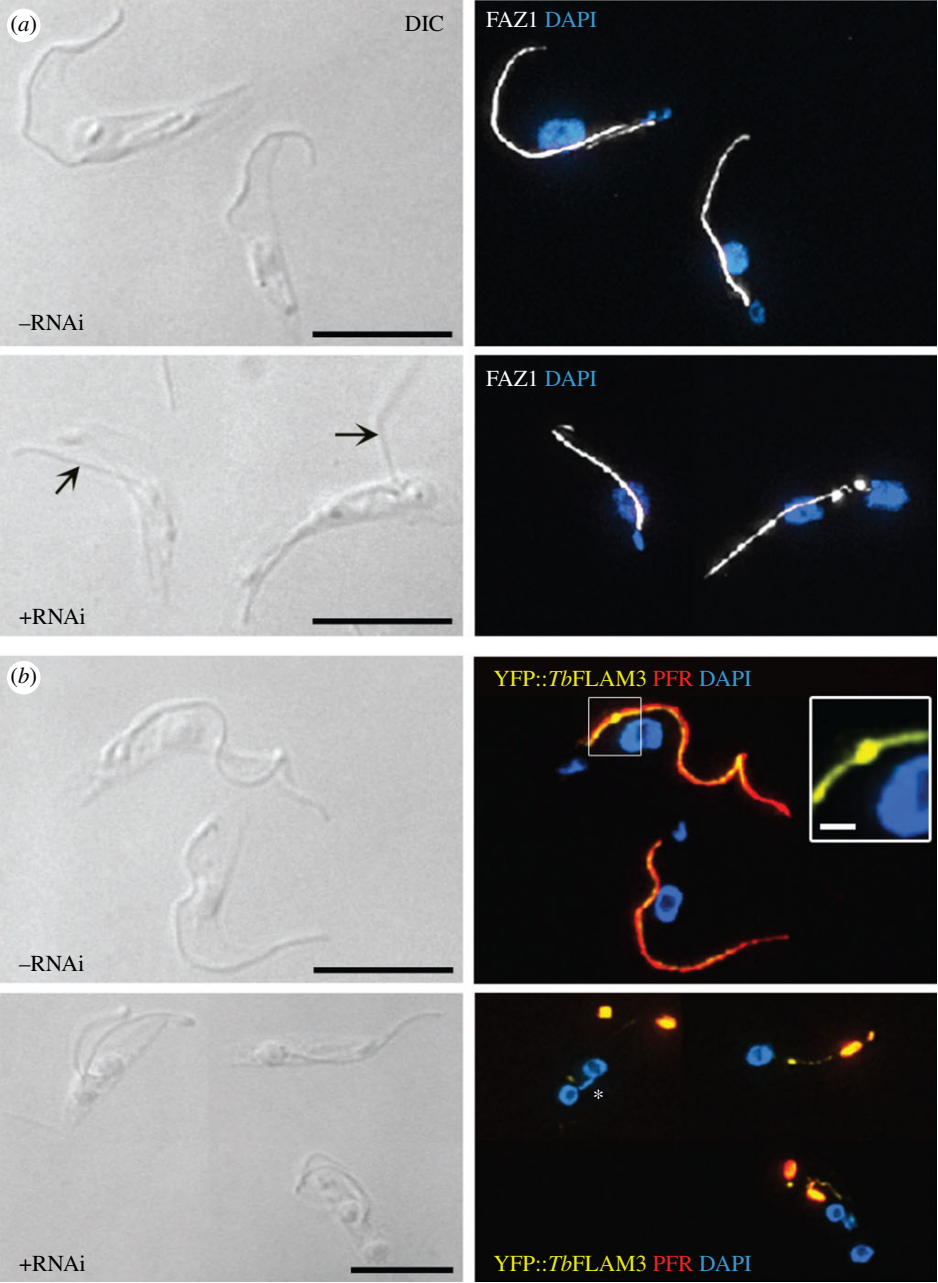
FAZ integrity following Tb*FOPL* RNAi. (*a*) Upper panel, normal FAZ1 localization on the intracellular face of the flagellum attachment zone (FAZ) in detergent-extracted cytoskeletons; lower panel, retention of FAZ1 localization following Tb*FOPL* RNAi and flagellum detachment. (*b*) Upper panel, normal YFP::*Tb*FLAM3 localization on the intraflagellar face of the FAZ in detergent-extracted cytoskeletons; lower panel, mis-localization of YFP::*Tb*FLAM3 following Tb*FOPL* RNAi. FAZ1 detected by monoclonal antibody L3B2, PFR was detected using monoclonal antibody L8C4. Scale bars in all main panels indicate 10 µm and in the inset panel 1 µm.

To interrogate further the observation of flagellum detachment from the cell body, we queried the localization of flagellum attachment zone (FAZ) components following Tb*FOPL* RNAi induction. On the cell body side of the FAZ, we observed a normal localization of FAZ1, a component of the fibres radiating from membrane junctional complexes [46], even in the absence of flagellum attachment (figure 6*a*). This was consistent with initial flagellum–cell body attachment in cells where no PFR is built [22]. On the intraflagellar side of the FAZ, we queried the localization of the high molecular weight protein *Tb*FLAM3 [47,48]. Here, the normal localization was lost following RNAi induction, with YFP::FLAM3 co-localizing with the aberrant intraflagellar accumulation of PFR2 at the distal end of the cell body (figure 6*b*). In these experiments, detergent-extracted cytoskeletons, rather than intact cells, were examined; the retention of L8C4 signal and YFP fluorescence indicated a stable association of bulky, amorphous ‘PFR’ components with the cytoskeleton rather than the more labile accumulation of PFR components seen in some flagellar RNAi mutants [49].

### 3.3. Localization and flagellum exclusion of *Tb*FOPL::myc

**Figure 7.**
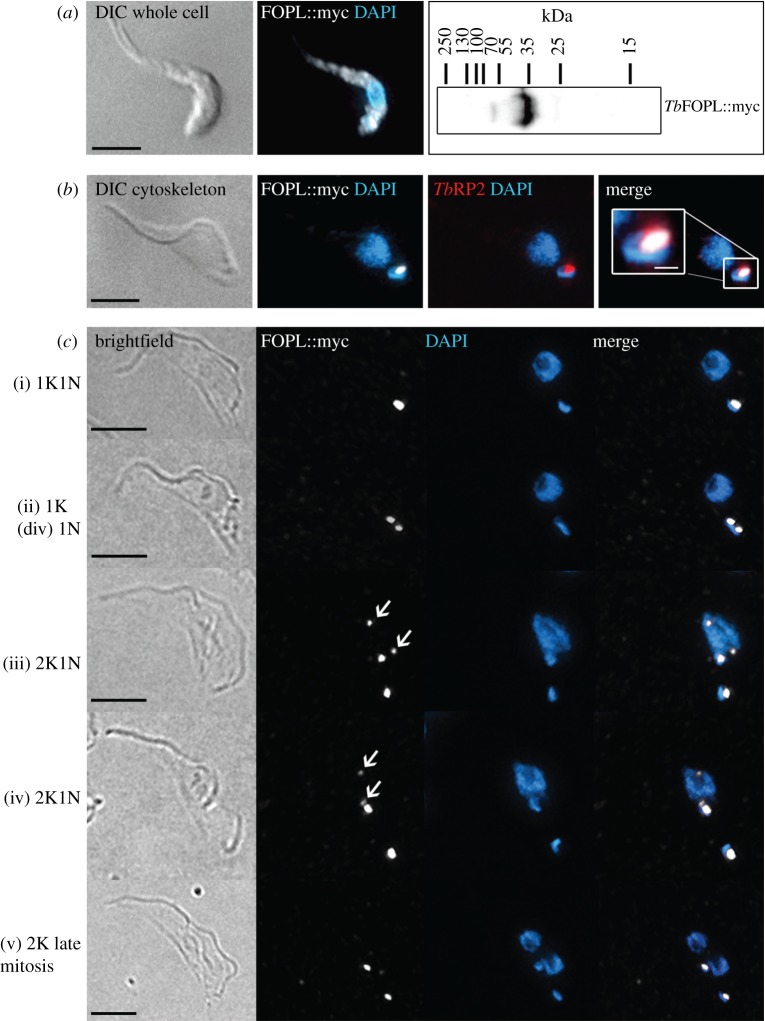
Localization of *Tb*FOPL::myc in procyclic *T. brucei.* (*a*) Basal body localization and cell body accumulation of *Tb*FOPL::myc in whole cells. The immunoblot confirms the expected molecular mass of *Tb*FOPL::myc; 5 × 10^6^ cell equivalents were loaded for SDS-PAGE. (*b*) Mature basal body localization of *Tb*FOPL::myc is retained in detergent-extracted cytoskeletons. (*c*) Additional localization of *Tb*FOPL::myc at spindle poles during early mitosis (arrows in cells (iii) and (iv)); images shown are detergent-extracted cytoskeletons. Scale bars in all main panels indicate 5 µm and in the inset panel 1 µm.

With the TONNEAU1 connection to acentriolar plant MTOCs and the microtubule-dominant organization of cell form in trypanosomes in mind, we questioned further the localization of *Tb*FOPL by expression of protein tagged at the C-terminus with GFP (*Tb*FOPL::GFP) and myc-tagged (*Tb*FOPL::myc) protein. *Tb*FOPL::GFP expressed from an endogenous locus, albeit under the regulation of a *PFR2* 3′ intergenic region, gave the same localization pattern as YFP::*Tb*FOPL. For *Tb*FOPL::myc, expression was driven from a strong RNA Polymerase I promoter. Over-expression of *Tb*FOPL::myc was evident from the accumulation of epitope-tagged protein in the cell body of whole cells, in addition to mature basal bodies (figure 7*a*). We included an over-expression of *Tb*FOPL to question why a basal body-localized protein was so critical for specifying correct assembly of an extra-axonemal structure approximately 2 µm distal to the basal body. Specifically, we considered whether there was a pool of intraflagellar *Tb*FOPL not seen when analysing the localization of fluorescent-tagged FOPL protein. There is indication that the transition zone limits protein access into the flagellum compartment on the basis of size (where only small proteins, less than 4.5 nm Stokes radii or 40 kDa, enter the flagellum by diffusion [50]). *Tb*FOPL::myc (predicted molecular mass approx. 30 kDa) fell comfortably beneath this threshold limit (cf. YFP-tagged *Tb*FOPL, molecular mass greater than 50 kDa). Notably, *Tb*FOPL::myc was excluded from the flagellum and the nucleus in whole cells (figure 7*a*). This leaves open the question of how a basal body-localized protein, *Tb*FOPL, critically influences the asymmetric assembly of the extra-axonemal PFR, which is built only after the flagellum exits its flagellar pocket, a distance of approximately 2 µm from the point where the axoneme initially extends from its basal body. Curiously, careful examination of detergent-extracted cytoskeletons revealed *Tb*FOPL::myc localization at the poles of the mitotic spindle in early mitotic cells (figure 7*c*(iii,iv)). This was in addition to basal body localization throughout the cell cycle. However, the nuclear signal was absent from cells fixed later in mitosis (figure 7*c*(v)). Re-examination of YFP::*Tb*FOPL localization did not reveal any indication of nuclear localization. The severe morphological abnormalities of *Tb*FOPL RNAi-induced cells meant it was not realistic to sensibly ascertain whether spindle formation was also compromised by an absence of *Tb*FOPL protein.

## 4. Discussion

The combination of N-terminal localized TOF-LisH motifs is a seldom used but highly effective means of localizing proteins to MTOCs: FOP family proteins including FOR20 and OFD1 are centriolar proteins conserved in diverse eukaryotes, and in humans, chromosomal translocation results in TOF-LisH-dependent retargeting of the tyrosine kinase domain of the FGFR1 receptor to the centrosome, and an atypical myeloproliferative disorder [1,2].

In trypanosomatids, and their free-living relative *Bodo saltans,* the presence of N-terminal TOF-LisH motifs in the GTPase activating protein RP2 provides a lineage-specific elaboration within the FOP family, and ensures basal body localization of the protein (in animals, basal body localization of RP2 is dependent on N-terminal acylation [26]). Curiously, although the evolutionary context is for N-terminal TOF-LisH motifs in eukaryotes, the single exception that we found in our bioinformatics analysis was of a gene model encoding a 688 amino acid protein with a candidate C-terminal TOF-LisH motif combination in the centric diatom *Thalassiosira pseudonana* (accession no. XP_002286300.1)*.* In all four trypanosome proteins that use a TOF-LisH motif combination, N-terminal fusion to YFP does not compromise localization to mature basal bodies, or in the case of *Tb*FOR20 localization to mature and associated pro-basal bodies [4]. Here, the more significant and unanticipated characteristic of the trypanosome FOP protein family is that *Tb*FOPL is not required for axoneme elongation but is essential for the assembly of extra-axonemal PFR. This contrasts strikingly with the essentiality of the mammalian FOP homologue at the earliest stages of ciliogenesis [6,7].

Similar to the *T. brucei* calmodulin (CaM) RNAi mutant, where PFR assembly is also totally compromised [22], a failure of PFR assembly in the *Tb*FOPL-depleted cells leads to flagellum–cell body detachment and abnormalities in cell morphogenesis. However, *Tb*CaM is present in both the PFR lattice and struts linking the PFR and outer doublet microtubules of the axoneme. Thus, the *Tb*CaM PFR assembly defect can be readily explained. By contrast, *Tb*FOPL appears not to be present within the flagellum compartment, even when expressed from a strong transcription promoter, thus raising the question as to how a basal body located protein is essential for PFR assembly.

We found no indication of problems in axonemal assembly within Tb*FOPL*-depleted trypanosomes—flagellum length was normal and only seldom was there a discernible defect in axoneme ultrastructure. This indicated that IFT was not lost, but rather that *Tb*FOPL deficiency caused a specific defect in PFR assembly. PFR assembly apparently initiates in some Tb*FOPL* RNAi-induced cells but then subsequently fails: PFR material accumulates as large unstructured deposits within or at the end of the flagellum in a majority of cells (approx. 96% of flagella 48 h post-RNAi induction in which PFR2 could be detected by immunofluorescence using monoclonal antibody L8C4; at 24 h post-RNAi induction, approximately 60% of flagella showed abnormal accumulation of PFR2). Basal body-localized *Tb*FOPL could play a critical, direct role in the import of PFR-specific cargo into the flagellum (i.e. although major components such as PFR1, PFR2 and FLAM3 are imported into the flagellum, it is possible that not all PFR components are imported thereby compromising intraflagellar assembly of the PFR lattice). In this context, there is precedent for selective transport of axonemal subcomplexes and/or roles for cytoplasmic chaperones or other accessory assembly proteins in pre-assembly of a variety of proteins associated with distinct axonemal substructures (e.g. dynein arms) prior to subcomplex import into the flagellum [51–54]. Indeed, in immunofluorescence experiments using monoclonal antibody ROD1, which recognizes an antigen from the outer or most distal region of the PFR lattice, signal intensity was severely reduced on some cells or non-existent in others (data not shown); this was reminiscent of similar immunofluorescence experiments in PFR-deficient *snl*-mutants [49].

Alternatively, the domain architecture of *Tb*FOPL is not typically reflective of either an enzyme or a chaperone. Rather, its architecture and available experimental evidence are more consistent with roles in scaffolding or mediation of protein–protein interaction. For instance, in mammalian cells, FOP recruits the centrosomal protein CEP19, which in turn interacts with the GTPase RABL2, and it is RABL2 that regulates IFT-B function and thereby cilium assembly [55]. Similarly, centrosome-localized FOP is required for anchoring microtubules to subcellular structures and localization of the centrosomal protein EB1, a plus-end microtubule-binding protein that has critical functions in regulating + end microtubule dynamics [56]. Notwithstanding the possibility that the filament-like PFR lattice could be self-assembling rather than chaperone- or accessory protein-dependent, *Tb*FOPL could feasibly play a role in recruiting another protein or proteins that act in PFR assembly and/or intraflagellar PFR attachment. At this point, our data are consistent with a direct or an indirect role for *Tb*FOPL in PFR assembly.

Presently, a final possibility to consider regarding how *Tb*FOPL influences PFR assembly is a possible dependency and/or interaction between *Tb*FOPL and *Tb*KIF9B, a trypanosomatid-specific, basal body- and axoneme-localized kinesin required for normal PFR construction [57]. Although there is similarity between the RNAi phenotypes of Tb*FOPL* and Tb*KIF9B*, the KIF9B phenotype is unique in that in a majority of cells, a PFR forms in patches along the length of the flagellum; in other cells, a PFR is either absent or accumulates in a single patch. Whether it is basal body- and/or axoneme-localized KIF9B that is required for normal PFR construction is not known [57]. Probing for potential interaction between these proteins represents one avenue with which to move forwards to understand the mechanism by which *Tb*FOPL defines PFR formation.

Additional to understanding how a trilaminar PFR lattice assembles, there is also the specification of attachment of the PFR proximal region to outer doublets 4–7 of the axoneme. Cues that define the asymmetric attachment of PFR to axoneme at a position several microns distant from the basal body are unknown. Although structural asymmetries exist within basal/probasal bodies (e.g. [40,58,59]), it is difficult to understand how a centriolar-located protein such as *Tb*FOPL could influence the asymmetric attachment of the PFR to the axoneme. We note, however, that in the ciliate *Tetrahymena*, an FOP1-like protein (and polyglutamylated tubulin) is asymmetrically distributed around the basal body; it is proposed asymmetric distribution of the FOP1-like protein and polyglutamylated tubulin may stabilize basal bodies against mechanical forces generated during ciliary beating [40]. From our current DeltaVision imaging, we have been unable to detect asymmetric localization of *Tb*FOPL or polyglutamylated tubulin (using the anti-polyglutamylation monoclonal antibody GT335) at the *T. brucei* basal body, but nevertheless, the *Tetrahymena* example raises the possibility that asymmetric distribution of basal body-localized proteins, such as FOP, could, either directly or via post-translational modification of axonemal microtubules, affect PFR attachment.

We find the possible nuclear localization of *Tb*FOPL::myc in early mitotic cells intriguing. Promoter-driven expression of *Tb*FOPL::myc occurs throughout the cell cycle but nuclear acquisition of *Tb*FOPL::myc is cell cycle dependent. We are therefore inclined to believe recruitment to the nucleus (and potentially to the MTOCs nucleating the mitotic spindle) is genuine. Although no discrete structures, such as centrosomes or spindle pole bodies (seen in yeast cells), have been observed in *T. brucei*, distinct ring-like structures, that appear to nucleate spindle microtubules, can be visualized by electron microscopy (reviewed in [60]). Our observation of nuclear recruitment of *Tb*FOPL indicates that in addition to a critical basal body-related function, the protein may also be required to establish the acentriolar MTOCs responsible for spindle microtubule nucleation. This observation potentially provides a parallel with the acentriolar MTOC localization of another FOP family protein, TONNEAU1, in plants [13]. We have not observed nuclear recruitment of FOR20 and T*b*RP2 (i.e. other trypanosome TOF-LisH proteins) even after over-expression (data not shown), and so recruitment of *Tb*FOPL to a nuclear MTOC does not appear to be a general feature of TOF-LisH targeting in trypanosomatids. In common with many unicellular eukaryotes, *T. brucei* undergoes a closed mitosis [61] and proteins involved in mitosis must be transported across the nuclear envelope. However, any possibility *Tb*FOPL is involved in MTOC function other than at basal bodies should be balanced with the observation that, like other known trypanosomatid basal body proteins, a *Tb*FOPL orthologue is absent from *Perkinsela*, an acentriolar, aflagellate basal kinetoplastid that is an endosymbiont within *Paramoeba* isolates [62]. This could imply that *Tb*FOPL is either not essential for spindle assembly and/or function in trypanosomes or that mitosis in *Perkinsela* occurs independently of FOPL.

In this, and previous work [4,24], we have investigated all four TOF-LisH motif-containing proteins expressed in *T. brucei*. All four proteins localize to the mature basal body. *Tb*FOR20 additionally locates to the probasal body and *Tb*FOPL potentially to mitotic spindle poles. *Tb*FOPL, *Tb*OFD1 and *Tb*RP2 have distinct flagellum assembly-related functions, but no apparent phenotype is detected in FOR20-depleted cells. We have previously shown that RNAi-mediated knockdown of *Tb*RP2 affects flagellum assembly and it is proposed that *Tb*RP2 acts as a GTPase activating protein with a role in protein trafficking; human RP2 acts as a GAP for the small GTPase ARL3 [63]. In *T. brucei* cells depleted for OFD1, the short flagellum phenotype generated suggests that IFT-mediated transport may be compromised in these cells, consistent with a proposed IFT-related role for mammalian OFD1. By contrast, the protein we have identified as being most similar to mammalian FOP appears to have a distinctive phenotype relating to PFR assembly, but without affecting axoneme assembly. Studying FOP-like function in trypanosomes affords a unique opportunity to study assembly of extra-axonemal structures. Finally, although the PFR is unique to trypanosomes and evolutionary close relatives, extra-axonemal structures are observed in flagella in a diversity of flagellated eukaryotes, including *Giardia intestinalis, Gymnodinium aureolum* and other dinoflagellates. There are also unusual MTOCs, some of which are thought to have a flagellar origin (e.g. the apical polar ring of apicomplexans and their near relatives [64–67]). Determination of whether FOP-related or other FOP family proteins play roles in the assembly or function of these cytoskeletal structures offers intriguing possibilities for future research.

## 5. Notes added in revision

Reference to TrypTag.org, the genome-wide project to localize every protein-coding gene-product in *T. brucei* [68], gives an indication of basal body localization for N-terminally mNeonGreen-tagged *Tb*FOPL, but there are currently no localization data available for *Tb*OFD1. mNeonGreen::*Tb*KIF9B localization does not mirror precisely the published localization: an additional, cell-cycle-stage-specific flagellar tip signal is reported alongside localization to pro- and mature basal bodies. This potentially adds further complexity to understanding the mechanistic basis for KIF9B-dependent PFR assembly [57] or any hypothetical interaction or dependency between KIF9B and FOPL.

## Supplementary Material

Supplementary Figure 1. Localisation of YFP::TbOFD1 in procyclic T. brucei; Supplementary Figure 2. Orthology of Tb927.10.3000 and HsOFD1; Supplementary Figure 3. Penetrance of TbFOP RNAi

## Data accessibility

Datasets supporting this article are provided in the electronic supplementary material.
