# Supplementary material for: A centriolar FGR1 oncogene partner-like protein required for paraflagellar rod assembly, but not axoneme assembly in African trypanosomes
Source: Open Biol. 2018 Jul 25;8(7):170218. doi: 10.1098/rsob.170218 (PMC6070722; doi:10.1098/rsob.170218)
Supplement: Supplementary Figure 1. Localisation of YFP::TbOFD1 in procyclic T. brucei; Supplementary Figure 2. Orthology of Tb927.10.3000 and HsOFD1; Supplementary Figure 3. Penetrance of TbFOP RNAi [file rsob170218supp1.pdf]

A centriolar FGR oncogene partner-like protein required for paraflagellar rod assembly, but not axoneme assembly in African trypanosomes

Jane Harmer[1,4], Katie Towers[2], Max Addison[1], Sue Vaughan[2], Michael L. Ginger[3*], Paul G. McKean[1*]

[1]Faculty of Health and Medicine, Division of Biomedical and Life Sciences, Lancaster University, Lancaster, LA1 4YQ, UK
[2]Department of Biological and Medical Sciences, Faculty of Health and Life Science, Oxford Brookes University, Gipsy Lane, Oxford, OX3 0BP, UK
[3]Department of Biological and Geographical Sciences, School of Applied Sciences, University of Huddersfield, Queensgate, Huddersfield, HD1 3DH, UK
[4]Present address: Department of Biological and Geographical Sciences, School of Applied Sciences, University of Huddersfield, Queensgate, Huddersfield, HD1 3DH, UK

Authors for correspondence:
Michael L. Ginger email: M.Ginger@hud.ac.uk
Paul G. McKean email: p.mckean@lancaster.ac.uk

Electronic Supplemental Information

Supplementary Figure 1. Localisation of Tb927.10.3000 gene product in procyclic *T. brucei*. (**A-B**) YFP::*Tb*927.10.3000 is present at mature basal bodies throughout procyclic cell cycle. Monoclonal antibody YL1/2 detected tyrosinated α-tubulin prominent on subpellicular microtubules at the posterior pole of whole cells and *Tb*RP2 (Reference 4 in the main text) at the mature basal body. Scale bars in all main panels indicate 5 µm and in the inset panels 1 µm.

Supplementary Figure 2. Orthology of Tb927.10.3000 and *Hs*OFD1. (**A**) Cartoon representation of human and *T. brucei* OFD1 proteins, showing insertions necessary to achieve maximal alignment of amino acid sequences. (**B**) Amino acid alignment of *Homo sapiens* (Accession number AC003037.1) and *T. brucei* (Tb927.10.3000) OFD1. (**C**) Effect of *Tb*927.10.3000 RNAi induction on trypanosome growth (triangles; solid line) compared to RNAi non-induced controls (diamonds; dashed line); immunoblotting with monoclonal antibody BB2 (detecting an N-terminal Ty-epitope) indicated depletion of YFP::*Tb*927.10.3000 post-RNAi induction. Polyclonal rabbit sera detecting trypanosome adenylate kinase isoform F (Ginger ML et al. 2005 *J. Biol. Chem.* **280**, 11781-9 doi: 10.1074/jbc.M413821200) was used as a loading control. (**D**) Preliminary analysis of cell morphology following *Tb*927.10.3000 RNAi induction: cells were scored for normal morphology versus assembly of an abnormal, short flagellum (at 24 h post-RNAi induction n = 51 'non-induced' cells / n = 67 'induced' cells; 48 h post-induction n = 41 'non-induced' / n = 75 'induced'; 72 h post-induction n = 39 'non-induced' / n = 95 'induced'; 96 h post-induction n = 25 'non-induced' / n = 123 'induced'). (**E-M**) Electron and fluorescence microscopy analysis of cell morphology: E-G, TEM analysis illustrating in short flagella the accumulation of electron dense material, potentially including unassembled PFR components, around normal 9+2 axoneme architecture; H-J, SEM analysis illustrating the short flagellum phenotype of *Tb*927.10.3000 RNAi mutants; K-L, fluorescence microscopy illustrating mixed 'short flagellum' and 'short cell' phenotypes in *Tb*927.10.3000 RNAi mutants (96 h post-RNAi induction); M, normal cell morphologies in *Tb*927.10.3000 cells not induced for RNAi against *Tb*927.10.3000. In K-M, the PFR is immunolabelled with monoclonal antibody L8C4 (red); DAPI (blue) was used to stain nuclear and kinetoplast DNA.

Assessment of OFD1 candidature. Mature basal body localisation of YFP::*Tb*927.10.3000 is analogous to mature centriole localisation of human OFD1 (Singla V et al. (2010) *Dev. Cell* **18**, 410-24 doi: 10.1016/j.devcel.2009.12.022). The short flagellum phenotype of the RNAi mutant resembled published *T. brucei* intraflagellar transport (IFT) RNAi phenotypes: the short flagellum phenotype of *Tb*927.10.3000 RNAi mutants is more similar in presentation to the phenotype arising from defective retrograde IFT than it is to defective anterograde IFT mutants, which fail to elongate an axoneme beyond the transition zone, and thus fail to build flagella (Absalon S et al. (2008) *Mol. Biol. Cell* **19**,

929-44 doi: 10.1091/mbc.E07-08-0749; Davidge J et al. (2006) *J. Cell Sci.* **119**, 3935-43 doi: 10.1242/jcs.03203). Flagellar membrane elongation or 'flagellar sleeve', seen in some *T. brucei* IFT mutants (Davidge et al. 2006), was also evident in SEM micrographs of *Tb*927.10.3000 RNAi-induced mutants. Loss of YFP::*Tb*927.10.3000 beneath the threshold of detection by immunoblot was evident 24 h post-RNAi induction, but presentation of the short flagellum phenotype was never evident across all cells in RNAi-induced cultures even by 96 h post-induction. We interpret such partial presentation of morphological phenotype, in contrast to the depletion of YFP::*Tb*927.10.3000 beneath a threshold level of detection, coupled to literature reports of OFD1 function in mammals, including a requirement in murine embryonic stem cells for OFD1 in formation of centriole distal-end appendages and IFT88 recruitment, to suggest that protein encoded by *Tb*927.10.3000 provides regulatory or indirect function(s) in IFT, rather than being a core part of the IFT machinery. In summary, candidature of *Tb*927.10.3000 as an OFD1 ortholog based only on amino acid sequence alignment with *Hs*OFD1 is equivocal, but coupled to comprehensive preliminary RNAi phenotype analysis evidence for OFD1 candidature is persuasive.

Supplementary Figure 3. Penetrance of *Tb*FOPL RNAi. Representative fields of view for whole cells show rapid loss of normal cell morphology and aberration of normal PFR biogenesis in populations at 24 (**A-C**) and 48 (**D-F**) h post-RNAi induction. Scale bars represent 10 μm.

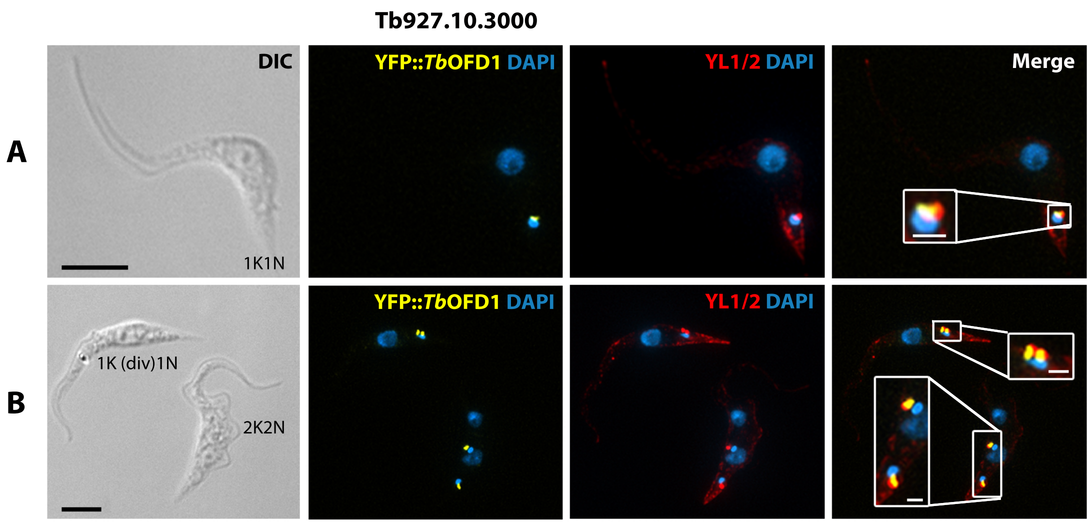

Supplementary Fig. 1

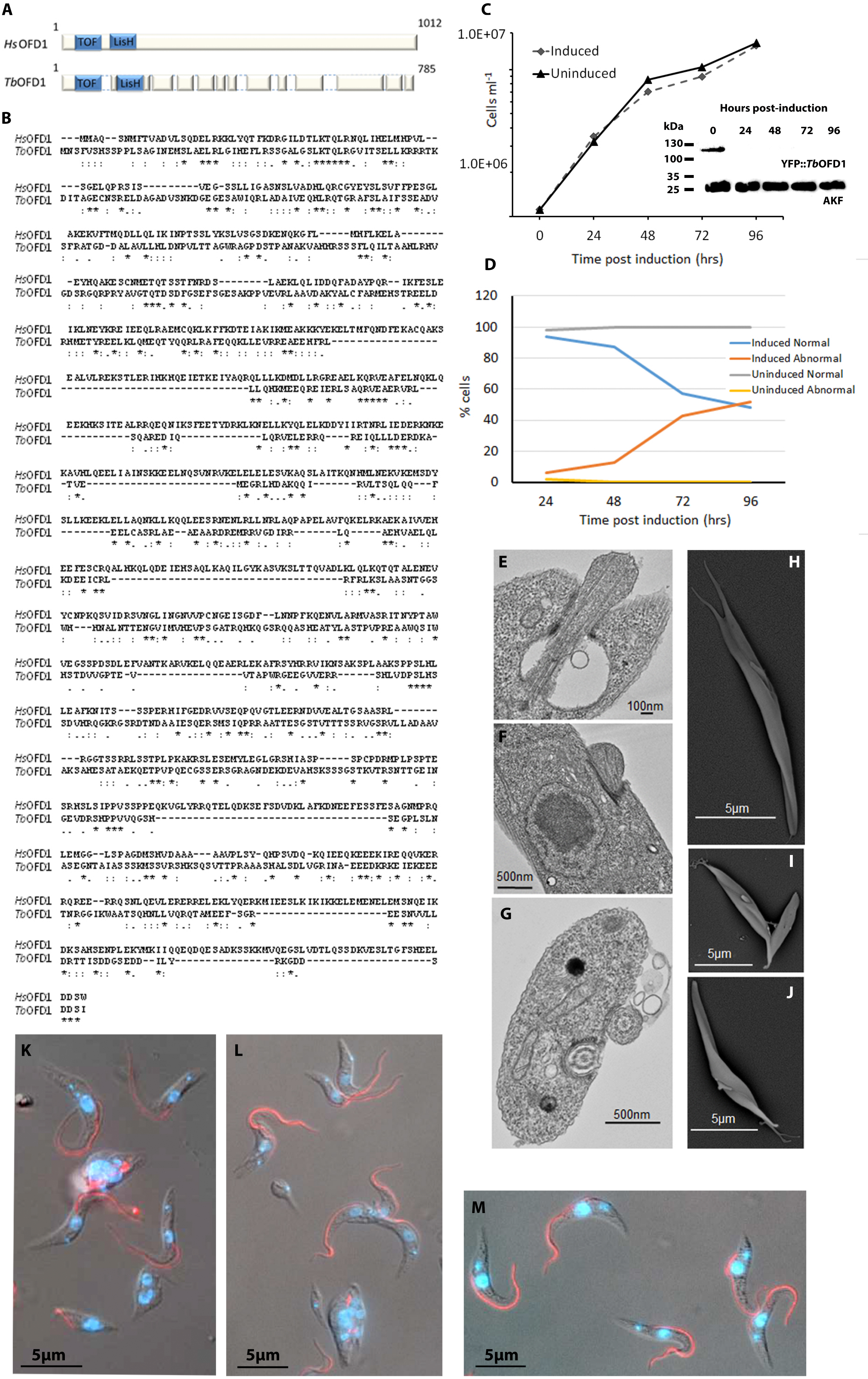

Supplementary Fig. 2

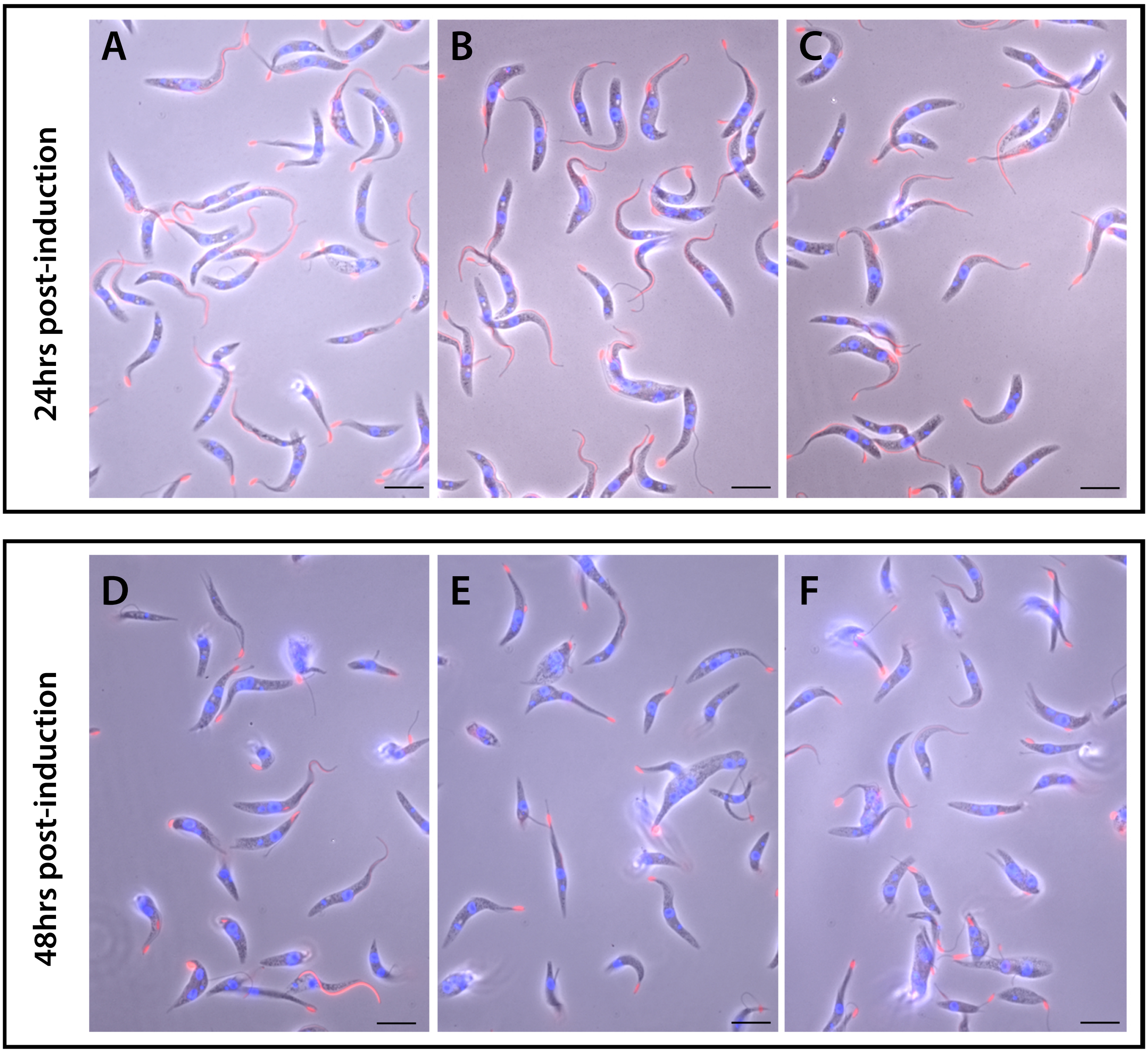

Supplementary Fig. 3
